# Unveiling CCL5: the master regulator of breast cancer progression and immune evasion

**DOI:** 10.1186/s13058-026-02364-y

**Published:** 2026-08-06

**Authors:** JiaYing Mu, MeiZhi Zhang, Qian Chen, WeiDong Liu, WeiKe Feng, YaNan Zhang

**Affiliations:** 1 College of Traditional Chinese Medicine, Shandong University of Traditional Chinese Medicine, Jinan, 250355 Shandong Province China; 2 Medical College, Shandong University of Traditional Chinese Medicine, Jinan, 250355 Shandong Province China

**Keywords:** CCL5, Breast cancer, Proliferation, Metastasis, Immune microenvironment

## Abstract

CCL5, abbreviated from C–C motif chemokine ligand 5, exerts diverse regulatory effects on both the initiation and progression of breast cancer (BC), making it a focal point of extensive research within the field. By enhancing the growth, invasive ability, and metastatic potential of BC cells, simultaneously recruiting and polarizing immunosuppressive cell populations, CCL5 actively reshapes the tumor immune microenvironment. Furthermore, elevated CCL5 levels are strongly linked to particular clinicopathological parameters, worse clinical outcomes, and a higher likelihood of recurrence in BC, suggesting its value in early diagnosis and prognosis. Current preclinical studies targeting the CCL5/CCR5 axis have demonstrated notable efficacy in inhibiting the progression of BC. This article explores how CCL5 contributes to BC progression and elucidates its regulatory mechanisms, providing theoretical support and potential targets for innovative therapeutic approaches.

## Introduction

An upward trend has been observed in breast cancer (BC) occurrence. Recent estimates suggest that by 2050, global BC incidence will rise to 3.2 million, accompanied by 1.1 million BC-associated death, presenting an increasingly severe challenge for prevention and control [1]. Despite the significant role of targeted therapies and immunotherapy in improving the survival rates among those diagnosed with BC, the onset and progression of BC remain major challenges in clinical treatment. Therefore, continued and comprehensive investigations of the biological processes driving BC formation are of crucial importance for optimizing current treatment approaches and developing new therapeutic strategies.

Recent years have seen growing interest in the role chemokines that play in initiating and advancing BC. Nearly 50 endogenous chemokines have been identified, which include the CXC, CC, CX3C, and C families. These chemokines exert their effects by interacting with specific receptors located on the cell membrane—7 TM-domain G protein-coupled receptors (GPCRs) [2]—which activate chemokine receptors, triggering a cascade of downstream effector activations and signaling peptide reactions [3].

CCL5, also identified as RANTES within the C–C chemokine family, acts as an important regulator in BC development, promoting proliferation, spread, and recurrence of cancer cells. This paper investigates the functions and underlying mechanisms of CCL5 in BC progression, with an emphasis on its contributions to promoting proliferation, enhancing invasion and metastasis, remodeling the immune microenvironment, guiding early diagnosis, evaluating prognosis, and targeting the CCL5/CCR5 axis for therapeutic interventions, thereby offering potential targets for the development of novel BC treatment strategies.

## Biological characteristic of CCL5

CCL5 is secreted by NK cells [4], T cells, macrophages, cancer-associated fibroblasts (CAFs), and tumor cells [5]. Its molecular weight is 8 kDa, consisting of 68 amino acids [6]. The expression of CCL5 is influenced by inflammatory factors. Lin et al. [7] found that the persistent activation of CAFs through TNF-α and IL-1β significantly increased CCL5 secretion. Notably, the expression of CCL5 was also regulated by epigenetic factors. Research by Dangaj et al. [8] showed that BC cells reduced their CCL5 expression by silencing the CCL5 gene through DNA methylation, leading to lower local CCL5 levels in the tumor. This decrease limited effector T cell migration to the tumor area, impaired anti-tumor immunity, and facilitated immune evasion. Consequently, the tumor microenvironment becomed increasingly immunosuppressive, further promoting tumor growth, invasion, and metastasis.

CCL5 primarily interacts with C–C motif chemokine receptor 1(CCR1), C–C motif chemokine receptor 3(CCR3), C–C motif chemokine receptor 4(CCR4), and C–C motif chemokine receptor 5(CCR5) [9]. Additionally, CCL5 exerts its biological functions by interacting with several receptors, including atypical chemokine receptor 1 (ACKR1/DARC), atypical chemokine receptor 2 (ACKR2/D6) [10], and chemokine receptor-like protein 2 (CCRL2/CRAM-B) [11]. Among these receptors, CCR5 has the highest affinity for CCL5 [12]. CCR5 is broadly present on immune cell membranes, endothelial cells, and tumor cells [13]. Research has demonstrated that the CCL5/CCR5 axis engaged with pathways like phosphoinositide 3-kinase (PI3K)/ protein kinase B (Akt) and nuclear factor kappa-B (NF-κB) to establish a microenvironment conducive to tumor cells survival. This interaction promoted uncontrolled tumor cells growth and sustained survival capacity. Moreover, it regulated the expression of matrix metalloproteinases (MMPs), growth factors, and inflammatory cytokines, which further enhanced tumor cell metastasis and invasion.

Therefore, CCL5 binding to CCR5 is pivotal in shaping the tumor microenvironment, orchestrating a complex signaling network essential for tumor progression.

## CCL5-mediated breast cancer cell proliferation

A meta-analysis involving 3087 BC patients and 3735 control subjects [14] revealed a strong association between elevated levels of CCL5/CCR5 and BC susceptibility. Additionally, Soria et al. [15] observed high expression of both CCL5 and C–C motif chemokine ligand 2(CCL2) at the primary tumor sites in BC, while expression levels were substantially decreased in the non-cancerous epithelial ductal cells adjacent to the tumor cells. This suggests that the upregulation of CCL5 and CCL2 expression occurred in the course of malignant evolution in BC cells.

Nanog, a transcription factor closely linked to the proliferation, differentiation, invasion, and metastasis of malignant tumor cells, is an important marker for assessing the growth and invasiveness of BC stem cells in vitro [16]. Research by Jin et al. [17] found that overexpression of CCL5 was considered a risk factor for BC progression and inhibiting the expression of CCL5 in BC cells effectively reduced the Nanog expression. Furthermore, MTT assays confirmed that silencing CCL5 expression led to a marked reduction in cell proliferation rates, highlighting the critical involvement of CCL5 in promoting BC cells proliferation.

Huang [18] cultured the BC MDA-MB-231 cell line in conditioned medium (CM) from hepatic stellate cells (HSCs), specifically the LX-2 cell line. The proliferation of BC cells was evaluated with the CCK-8 assay. Results indicated that silencing CCL5 expression in HSCs via small interfering RNA (siRNA) significantly reduced the proliferative capacity of BC cells grown in co-culture conditions. Conversely, the addition of exogenous CCL5 to the CM increased the growth potential of the BC cells. Moreover, inhibiting the expression of CCR5 on the BC cells using an antagonist significantly suppressed their proliferation. These findings suggested that the CCL5 produced by HSCs bound to CCR5 receptors present on BC cells, thereby promoting their proliferation. The authors also performed bioinformatics analysis on the cytokines and found that CCL5-related signaling pathways were primarily enriched within the PI3K/Akt pathway. They proposed that, within the co-culture model, through engaging with PI3K/Akt pathway, the CCL5/CCR5 axis regulated the malignant behavior of BC cells.

In summary, CCL5 facilitates increased proliferation of BC cells. Its effect on cell proliferation mainly occurs through the upregulation of Nanog protein expression and upregulation of its receptor CCR5. In addition, it activates the PI3K/Akt signaling pathway and engages other associated molecular processes.

## CCL5 facilitating breast cancer invasion and metastasis

### Clinical evidence

Metastasis remains the leading cause of death in individuals with BC [19], and CCL5 is highly correlated with BC metastasis. The work of Qiu et al. [20] analyzed clinical data from 164 BC patients and found that CCL5 levels in the patients’ body were positively correlated with the axillary lymph node involvement, CCL5 expression in both serum and tumor tissue lysates was found to be positively correlated. Drawing upon datasets from the Gene Expression Omnibus (GEO, n = 1159) and The Cancer Genome Atlas (TCGA, n = 1084), the authors applied Weighted Gene Co-expression Network Analysis (WGCNA) to investigate the relationship between CCL5 and its corresponding receptors. The analysis revealed the strongest correlation between CCL5 and CCR5, suggesting that CCL5 may promote axillary lymph node metastasis in BC through CCR5.

An independent analysis [21] of a microarray dataset comprising 2254 BC samples from 27 studies revealed elevated expression of CCL5 and CCR5 in the basal-like and human epidermal growth factor receptor2 (HER2)-positive subtypes. These findings indicate that the CCL5/CCR5 axis tends to be more active in these specific BC categories. In addition, CCR5⁺ cells derived from the SUM-159 basal-like cell line demonstrated enhanced invasiveness, implying that CCR5 contributes to metastatic progression in basal-like and HER2-positive BC.

### Experimental findings

In addition to clinical research, numerous experimental studies have also found a close relationship between CCL5 and BC metastasis, with increased expression of CCR5 during BC development and metastasis [22]. Evidence from research indicated that in invasive BC, the expression of CCR5 is upregulated, leading to the migration of BC cells to metastatic sites [23]. CCR5 expressed on BC cells in response to CCL5 stimulation enhances the invasive potential of these cells [24].

Wang et al. [25] found that during the process of bone metastasis in BC, when BC cells reach the bones, receptor activator of NF-κB ligand (RANKL) within the bone microenvironment induced the expression of lymphotoxin-β (LTβ) in tumor cells. LTβ bound to LTβ receptors (LTβR) on osteoblasts, stimulating NF-κB p52 protein expression inside osteoblasts, thereby activating the NF-κB2 signaling pathway. This induced the production of chemokines CCL2 and CCL5. Through feedback, CCL2 and CCL5 recruited Raw264.7 macrophage cells and enhanced osteoclast differentiation, promoting the seeding of BC cells within the bone microenvironments and advancing osteolytic bone metastasis.

Zhang et al. [26] found that after Staphylococcus aureus (S. aureus) invaded BC cells, it activated NF-κB, which promoted expression of CCL5 downstream of NF-κB in the BC cells. In scratch assays, they observed that silencing CCL5 and CCR5 expression significantly reduced the wound healing rate of BC cells in comparison with those only invaded by S. aureus. This suggested that silencing CCL5 and CCR5 expression significantly impaired migration of BC cells, indicating that after S. aureus invasion, the CCL5/CCR5 axis influences the migration function of BC cells.

Zhao et al. [27] observed that S. aureus invasion enhances BC cell migration through the CCL5/CCR5 axis. S. aureus activated NF-κB p65 through RIG-I-like receptor signaling, further enhancing the expression of CCL5 and CCR5. Scratch assays revealed that after S. aureus invasion, the healing rate of BC cells significantly increased, while the healing rate of cells with silenced CCL5 expression or blocked CCR5 expression was notably reduced. These results demonstrated that S. aureus enhanced the level of CCL5 expressed by BC cells, by binding to CCR5, then enhanced the migration capacity of these cells.

#### CCL5/CCR5 axis and EMT

Tumor metastasis largely relied on epithelial–mesenchymal transition (EMT), during which epithelial cells acquire mesenchymal traits that increase their motility [28]. Liu et al. [29] conducted an in vitro study on the effects of sulfur mustard exposure, which significantly enhanced CCL5 secretion and the activation of the CCL5/CCR5 axis in MCF-7 BC cells. This exposure also upregulated Akt and p85α phosphorylation, thereby activating the PI3K/Akt pathway. Consequently, the epithelial marker E-cadherin (CDH1) were downregulated, while mesenchymal marker FN mRNA, along with SNAIL2 and vimentin, were upregulated, triggering EMT and promoting MCF-7 cells’ capabilities to migrate and invade.

Lin et al. [30] demonstrated that in a co-culture system of BC cells and tumor-associated macrophages (TAMs), lactate secreted by BC cells acted on Notch1/Jagged2 receptors on the surface of TAMs. This led to a marked upregulation of the Notch intracellular domain (NICD), subsequently enhancing CCL5 and transforming growth factor β(TGF-β) release by TAMs. Additionally, TGF-β further stimulated macrophages to secrete CCL5. CCL5, by binding to the CCR5 receptor on BC cells, upregulated the phosphorylation level of AMP-activated protein kinase (AMPK) in the cells, thereby promoting aerobic glycolysis and increasing lactate production, creating a positive feedback loop. This loop led to the downregulation of the epithelial marker E-cadherin accompanied by increased expression of mesenchymal markers vimentin and N-cadherin, suggesting that EMT had occurred in the BC cells. When the mixed-culture system was injected into tail veins of mice, lung metastatic lesions were detected upon dissection. Immunohistochemistry showed elevated levels of CCR5, hexokinase 2 (HK2), and phosphorylated AMPK (p-AMPK) within these lesions. Addition of a neutralizing antibody targeting CCL5 to the mixed-culture system not only suppressed BC lung metastasis but also lowered the expression of CCR5, HK2, and p-AMPK. These findings indicated that macrophage-derived CCL5 promoted EMT in BC cells and markedly increased their metastatic potential.

#### Additional mechanisms

The involvement of the CCL5/CCR5 axis has also been suggested in enhancing tumor invasion by inducing angiogenesis and altering the extracellular matrix (ECM), recruiting stromal cells, and regulating metabolism [31].

Tumor invasion and migration are often accompanied by extensive angiogenesis. The neovasculature supplies essential nutrients and oxygen that sustain tumor expansion and progression [32]. Zeng et al. [33] demonstrated under both in vitro and in vivo conditions that the release of CCL2/CCL5 into the serum by CAFs mediated by lncSNHG5-ZNF281 substantially diminished the ZO-1 and Occludin levels in endothelial cells. This led to an increase in microvascular density (MVD), upregulation of p-P38 expression in lung tissue, and promotion of angiogenesis. Additionally, the permeability of lung capillaries was enhanced, facilitating the generation of the pre-metastatic niche (PMN) in the lung tissue. They also found that injection of inhibitors to inhibit the CCL2/CCR2 and CCL5/CCR5 signaling pathways significantly inhibited lung PMN angiogenesis and reduced vascular permeability.

Further studies have shown that CCL5 promotes the secretion of MMPs including MMP9, MMP13, and MMP14, which degrade ECM proteins. Scratch assays and transwell migration assays revealed that CCL5 enhanced scratch healing and increased the motility of BC cells. Conversely, the use of MMP inhibitors reduced both scratch healing and the number of migrating cells. These experiments demonstrated that CCL5 enhances the metastasis of BC cells by promoting MMP-mediated ECM degradation, thus accelerating tumor cell migration in BC [34, 35].

Elizabeth Brett et al. [36] found that in a co-culture system involving MDA-MB-231 BC cells, adipocyte stem cells, and skin-derived fibroblasts, high levels of CCL5 were secreted jointly by the adipose-derived stem cells and MDA-MB-231 cells. This elevated CCL5 level induced the production of type VI collagen, which promoted cancer progression. This collagen was primarily found in the matrix adjacent to non-cancerous cells, and it contributed to the invasive and metastatic behavior of BC cells.

Additionally, D’Esposito et al. [37] discovered that in a high-glucose environment, triple-negative breast cancer (TNBC) cells co-cultured with human-derived adipocytes exhibited higher motility and invasiveness. Their study included immunohistochemical analysis of 40 TNBC patients, which revealed positive expression of CCL5 in the tumor-adjacent adipose tissue. Such expression showed a strong association with lymphatic and distant metastases as well as unfavorable prognosis. They proposed that the immune response induced by CCL5 secreted by adipocytes in the tumor-adjacent adipose microenvironment significantly contributeds to promoting tumor metastasis in TNBC patients.

Combining in vivo and in vitro experiments, Gao et al. [38] found that a high-glucose environment promoted the secretion of CCL5 by adipocytes. CCL5 activates the AKT/mTOR pathway by promoting the phosphorylation of AKT, mTOR and GSK3β in breast cancer cells, thereby upregulating GLUT1 expression and enhancing glycolysis. This process significantly promoted glucose uptake and elevating glycolytic flux in BC cells, leading to increased ATP production and ultimately enhancing the invasive capacity of BC cells.

**Fig. 1:**
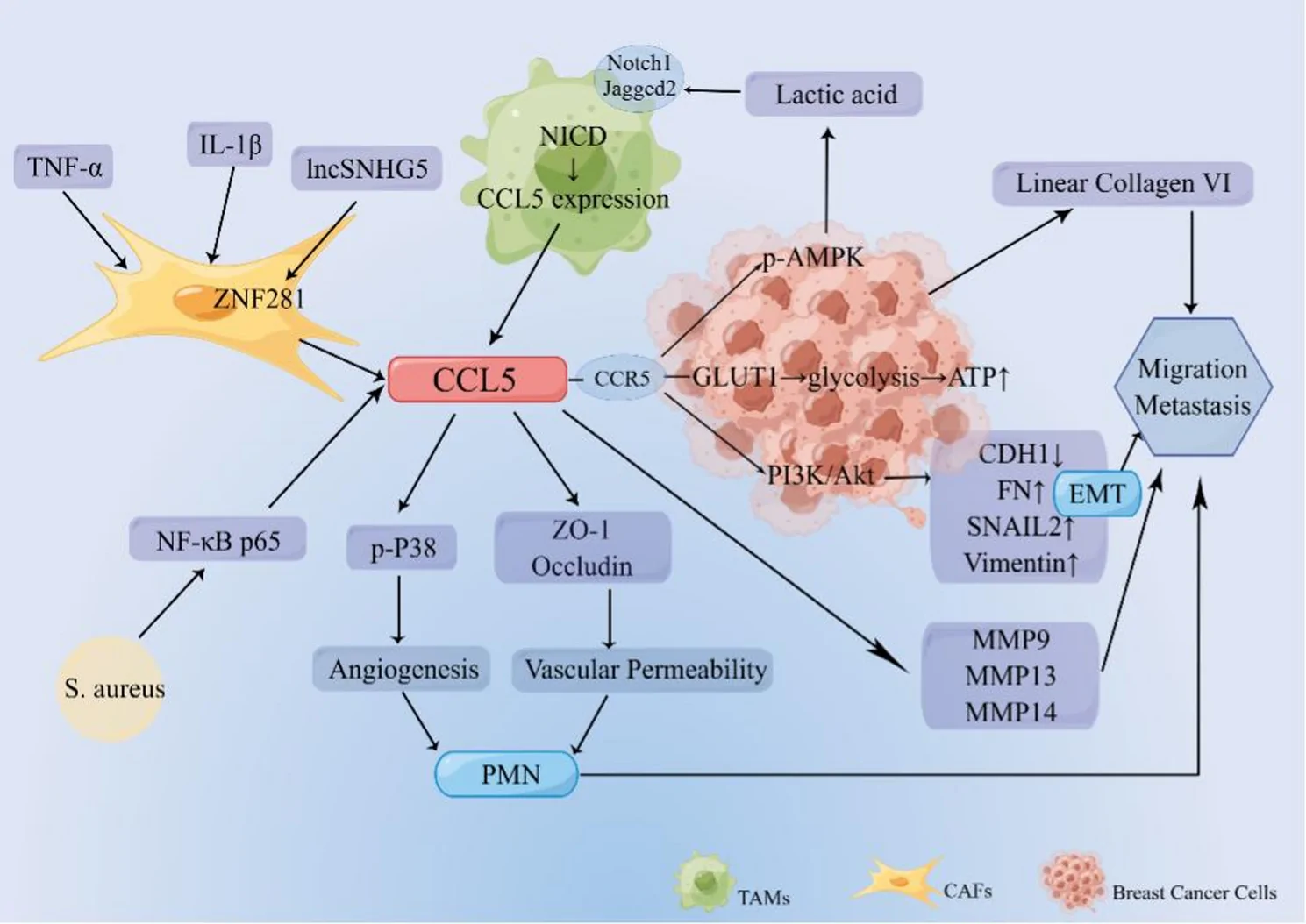
Mechanisms of CCL5 in promoting breast cancer proliferation, invasion, and metastasis. TAMs and CAFs secrete CCL5, which binds to CCR5 on breast cancer cells, activating PI3K/Akt, NF-κB, and p38 MAPK pathways. This signaling axis promotes epithelial–mesenchymal transition (EMT: downregulation of E-cadherin, upregulation of SNAIL2 and vimentin), enhances glycolysis via GLUT1-mediated glucose uptake, and induces matrix metalloproteinases (MMP9/13/14) to facilitate extracellular matrix degradation. CCL5 also stimulates linear collagen VI deposition, supporting tumor cell migration. Moreover, CCL5 compromises vascular integrity by reducing tight junction proteins (ZO-1, occludin), increasing permeability and promoting intravasation. Concurrently, it recruits polymorphonuclear neutrophils (PMNs), fostering an immunosuppressive and pro-metastatic microenvironment. Collectively, CCL5 integrates metabolic reprogramming, stromal remodeling, and immune modulation to accelerate breast cancer invasion and metastasis

In conclusion, CCL5 facilitates the metastatic spread and invasive behavior of BC by triggering the PI3K/Akt, AMPK, and NF-κB signaling cascades. It contributes to immune cell recruitment, EMT induction, extracellular matrix remodeling, angiogenesis promotion, metabolic regulation, and lymph node dissemination (Fig. 1).

## CCL5 in the breast cancer immune microenvironment

Various immune cell populations and the cytokines they release collectively make up the tumor immune microenvironment. Interactions between immune cells and tumor cells collectively influenced the progression of malignant tumors [39]. Research has shown that through the CCL5/CCR5 axis, cancer cells could recruit T cells, monocytes, bone marrow cells, fibroblasts, and mesenchymal stem cells (MSCs), and subsequently induce them to differentiate into immunosuppressive T regulatory cells (Tregs), TAMs (M2), myeloid-derived suppressor cells (MDSCs), and CAFs. This mechanism facilitated the formation of an immunosuppressive microenvironment, which in turn promoted tumor proliferation, invasion, and migration [40].

### CCL5 and macrophages

Within the tumor immune microenvironment, macrophages may polarize into two distinct phenotypes in response to different stimuli: M1 and M2 [41]. M1 macrophages produce multiple inflammatory cytokines such as IL-1β, IL-6, IL-12, TNF-α, and IL-23, playing a role in anti-tumor immunity. In contrast, M2 macrophages secrete anti-inflammatory cytokines such as IL-10, TGF-β, and IL-4, and highly express growth factors such as vascular endothelial growth factor (VEGF), epidermal growth factor (EGF), and fibroblast growth factor (FGF). These factors contribute to immune suppression and tumor promotion [42, 43].

CCL5 can induce the recruitment of macrophages and polarization towards the immunosuppressive M2 phenotype. Yamaguchi et al. [44] studied tissue samples from 111 BC patients and found that, in comparison with normal breast stromal cells, CCL5 was expressed in the stromal cells near BC cells. Furthermore, the immune reactivity of CCL5 was significantly correlated with the infiltration of CD163^+^ macrophages, indicating that CCL5 has a chemotactic effect on macrophages. Macrophage infiltration participates in BC progression and metastasis. Therefore, this study suggested that in BC tissue, CCL5 secreted by stromal cells might promote tumor progression by recruiting macrophages to the tumor area.

Zhu et al. [45] co-cultured the Luminal B-type BC ZR7530 cell line with the monocyte THP-1 cell line. They found that CCL5 secreted by BC cells, through binding to the CCR5 receptor on monocytes, promoted the phosphorylation of mitogen-activated protein kinase kinase 1/2 (MEK1/2), extracellular signal-regulated kinase 1/2 (ERK1/2), and signal transducer and activator of transcription 3 (STAT3) in the MEK/STAT3 signaling pathway. This further led to the upregulation of M2 marker proteins, such as CD163, CD206, Arg1 (Arginase-1), and IL-10, promoting the polarization of THP-1 monocytes into M2 macrophages. Nie et al. [46] studied the co-culture system of primary breast tumor (PT) cells and macrophages, finding that CCL5 secreted by PT cells, through binding to CCR5 on macrophages, significantly elevated the p-Akt level in TAMs. This Akt pathway activation resulted in elongated macrophage morphology, elevated CD206 expression, and reduced HLA-DR expression, consistent with TAMs polarizing to the M2 phenotype.

Zhang et al. [47] demonstrated that the zinc finger protein ZNF451, through Slug (Snail2)-mediated transcription of CCL5, recruited and activated TAMs, enhancing the expression of M2 macrophage markers such as CD206. This promoted M2 polarization of macrophages and accelerated the development of triple-negative BC. Frankenberger et al. [48] found that Raf kinase inhibitor protein (RKIP) reduced the several macrophage chemokines expression, including CCL5, by blocking HMGA2 signaling, inhibiting TAM recruitment. In RKIP^+^ BC cells, transfection with CCL5 increased its expression, which enhanced TAM recruitment. Additionally, using an antagonist to block CCR5 receptor significantly reduced TAM recruitment, suggesting that CCL5 can promote TAM recruitment and infiltration in RKIP^+^ BC cells.

### CCL5 and MDSCs

MDSCs originate from bone marrow progenitors and immature myeloid cells [49]. They continuously produce inflammatory cytokines and chemokines [50] and create a favorable immune microenvironment for tumor cell colonization and suppress NK cells, T cells, and other immune cell populations [51].

Studies have shown that CCL5 can promote the accumulation of MDSCs in tumors, enhancing their immunosuppressive functions [52]. Blattner et al. [53] found that the inflammatory cytokine IL-6 upregulated CCR5 expression in MDSCs via the STAT3 pathway. CCR5 promoted the recruitment and activation of MDSCs, and the presence of IL-6 induced a strong inhibitory effect on the function of CD8^+^ T cells by differentiated MDSCs. Gu et al. [54] found that when overexpressing and knocking down miR-9 in the BC 4T1 cell line and then inoculating it into mice, miR-9 inhibited SOCS3 expression and promoted CCL5 expression. Flow cytometry showed that miR-9 increased the proportion of granulocyte-like myeloid-derived suppressor cells (G-MDSCs) in the tumor tissue, suggesting miR-9 promoted the chemotactic enrichment of G-MDSCs in the tumor through CCL5/CCR5 signaling axis, thereby creating an immunosuppressive microenvironment that promotes BC proliferation.

### CCL5 and Tregs

Functioning as a distinct T cell subset, Tregs help maintain immune balance and tolerance, playing an important role in tumor immunity [55]. As immunosuppressive cells, Tregs inhibit effector T cell activity, thus diminishing the body’s anti-tumor immune response [56].

Rabe et al. [57] demonstrated that CCL5 regulated the secretion of tumor extracellular vesicles (EVs), which drived macrophages to adopt a pro-metastatic phenotype. These macrophages secreted CCL5, CXCL1, CCL19, and other chemokines, increasing the number of Foxp3^+^ Tregs in the tumor microenvironment (TME). Chang et al. [58] also found that CCL5 induced CD8^+^ T cell apoptosis and inhibited NK cell function by recruiting Tregs, thereby forming an immunosuppressive barrier. Qiu et al. [59] found that in BC patients with lymphatic metastasis, CCL5 levels in the tumor tissue were elevated, leading to increased expression of Treg markers CD25 and Foxp3. Flow cytometry revealed a significant increase in CD4^+^CD25^+^Foxp3^+^ Tregs in the tumor microenvironment, indicating that CCL5 facilitates the recruitment of Tregs to BC cells, thereby forming an immunosuppressive microenvironment.

### CCL5 and MSCs

CAFs are crucial in tumor progression [60] by interacting with tumor cells and immune cells to promote the malignant phenotype of tumors [61]. Mi et al. [62] found that tumor-derived osteopontin (OPN) interacted with the αvβ3 integrin on the surface of MSCs, activating the MSC transcription factor AP-1 and promoting CCL5 secretion. In turn, MSCs, under the influence of CCL5, secreted α-smooth muscle actin (α-SMA), tenascin-C, stromal cell-derived factor 1 (CXCL12, also known as SDF-1), and fibroblast-specific protein-1 (FSP-1), thus transforming into CAFs.

**Fig. 2:**
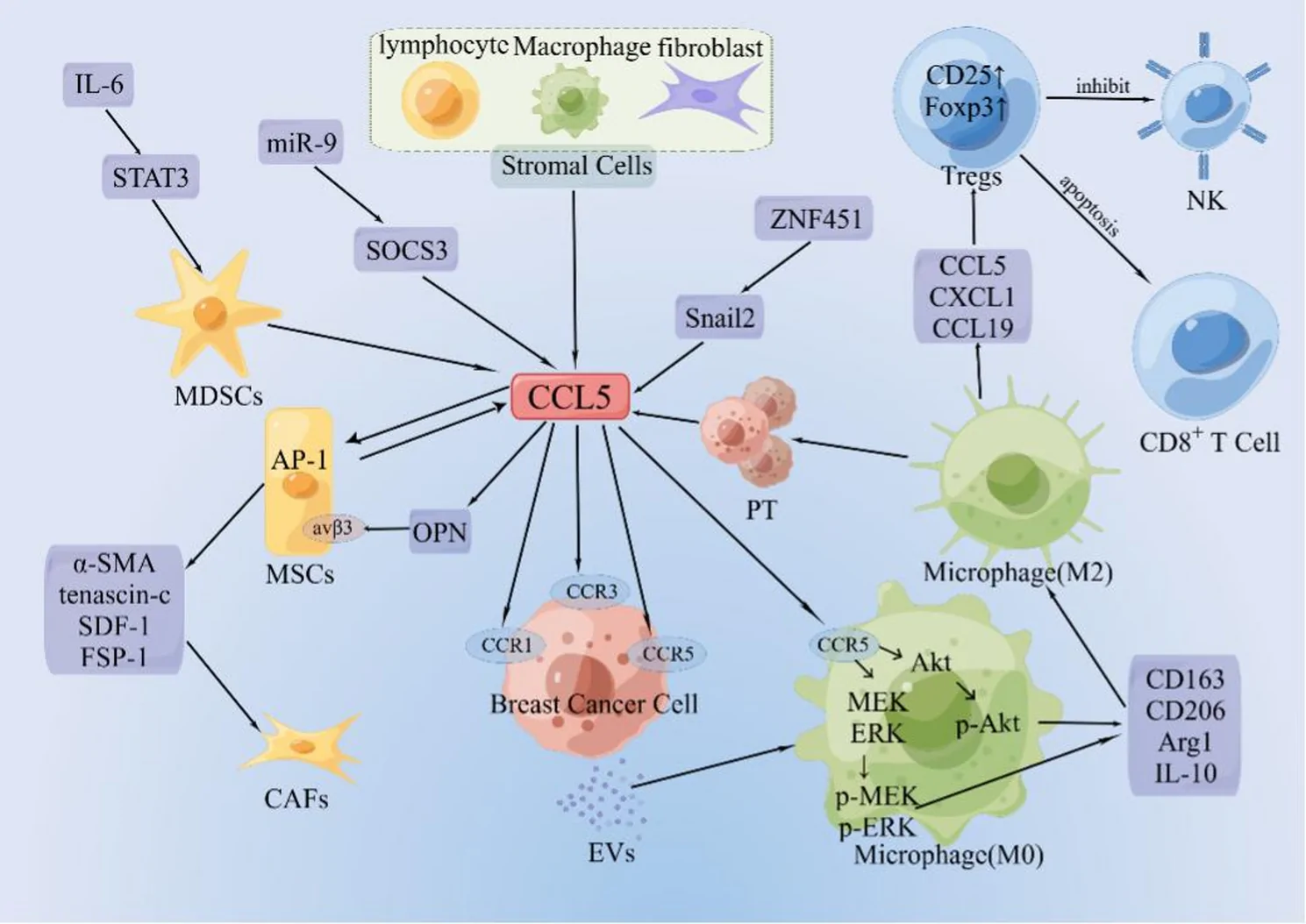
The role of CCL5 of BC in the tumor immune microenvironment. CCL5 is secreted by breast cancer cells and stromal cells (including lymphocytes, macrophages, and fibroblasts), promoting immune evasion and tumor progression. It binds to CCR3, CCR5, and CCR1 on breast cancer cells, activating Akt/MEK/ERK pathways to enhance proliferation and survival. CCL5 recruits and activates MDSCs via STAT3 and AP-1, driving expression of α-SMA, tenascin-c, SDF-1, and FSP-1—key markers of CAFs. In MSCs, CCL5 induces OPN secretion and αvβ3 integrin expression, further supporting CAFs differentiation. CCL5 also promotes M2 polarization of macrophages through CCR5-mediated Akt activation, increasing CD163, CD206, Arg1, and IL-10 expression. Additionally, CCL5 enhances Treg expansion (via ZNF451/Snail2) and secretion of CCL5, CXCL1, and CCL19, which inhibit NK cell function and induce CD8⁺ T cell apoptosis. EVs from tumor cells propagate CCL5 signaling, amplifying immunosuppression. Collectively, CCL5 remodels the tumor immune microenvironment toward immunosuppression and supports breast cancer progression

In summary, CCL5 shaped an immunosuppressive microenvironment favorable for BC progression by recruiting and polarizing TAMs through signaling pathways including MEK/STAT3, Akt, NF-κB p65/STAT3, and STAT3. CCL5 also recruited MDSCs Tregs, and induced MSCs to differentiate into CAFs (Fig. 2).

## CCL5 as a biomarker for breast cancer diagnosis, prognosis, and recurrence

Early diagnosis and precise prognostic evaluation are significant for improving the Early diagnosis and precise prognostic evaluation are significant for improving the patient survival rates in BC.

### Diagnostic potential

Early diagnosis helps detect BC at an early stage and significantly improves the survival rate of patients [63]. Hahoshen et al. [64] found that in comparison with stage I BC patients, CCL5 was expressed at higher levels in stage II BC patients. Therefore, high CCL5 expression, in combination with the lack of estrogen receptor (ER-α) expression, could serve as a biomarker for stage II BC.

Cancer antigen 15-3 (CA 15-3) is a pivotal biomarker for BC diagnosis and monitoring [65]. However, Dąbrowska et al. [66] conducted a study involving 100 patients with BC, and they found that BC patients exhibited markedly elevated plasma CCL5 levels but reduced CCR5 levels compared with healthy controls. Higher CCL5 expression was correlated with advanced tumor stages, whereas CCR5 levels declined as disease stage progressed. In addition, based on ROC curve analysis, the area under the curve of CCL5 was higher than the value corresponding to CA15-3. The AUC value of CCL5 significantly increased in the early stages of BC, and the maximum range observed in BC patients occurs when the measured indicators were combined with CA15-3 analysis. In summary, this study suggested that detecting CCL5 and CCR5 in plasma might have certain value in the diagnosis of the early stages of BC, especially when used in combination with CA15-3.

### Prognostic value

CCL5 also showed potential in the prognostic evaluation of inflammatory BC [67]. D’Esposito, Qiu, and others [37, 59] revealed a positive correlation between CCL5 and axillary lymph node metastasis in BC. CCL5 was highly expressed in patients with poor prognostic factors, such as ER-negative, PR-negative, higher nuclear grade, advanced clinical stage, and HER2 overexpression [20]. Collagen IV interacted with cell surface receptors such as integrins, facilitating the adhesion and motility of normal and transformed cells, and served as a key barrier to tumor cell metastasis [68]. Zhu et al. [69] analyzed 109 Luminal B (HER2-) invasive BC samples and identified a significant negative correlation between CCL5 and collagen IV. The CCL5/collagen IV ratio, as an independent prognostic indicator, effectively predicted 5-year disease-free survival, offering a novel approach to prognostic stratification in Luminal B (HER2-) BC.

### Recurrence

Further studies have indicated that CCL5 is also an effective target for preventing tumor recurrence [70]. Research indicated that some BC patients would experience recurrence 10–32 years after initial diagnosis. Among these, patients with a higher number of axillary lymph node metastases, larger tumor size, and estrogen receptor–positive BC had a higher likelihood of late recurrence (recurrence occurring ≥ 10 years after diagnosis) [71]. Yamaguchi et al. [44] found that BC patients with positive CCL5 and CCR3 expression but negative for CCR1 and CCR5 had a significantly higher risk of recurrence. They proposed that the CCL5/CCR3 axis was especially critical for BC recurrence, and that CCL5 secreted by stromal cells (lymphocytes, macrophages, fibroblasts) might activate CCR3 expressed on cancer cells, thereby controlling downstream mechanisms, including the ERK pathway in BC tissue, promoting tumor progression. Fazlollah Shokri et al. [72] found that in the treatment of TNBC, although radiation therapy effectively reduced the primary tumor volume, it also caused an increase in CCL5 concentration. This led to an elevation in monocyte levels in the blood, macrophage recruitment, infiltration, and polarization towards the immunosuppressive M2 subtype, thereby promoting tumor recurrence.

Additionally, Sandra Zazo et al. [73] discovered that CCL5 increased ERK phosphorylation, mediating acquired resistance of BC cells to trastuzumab. Moreover, continuous exposure of the drug-resistant BT-474 cells to trastuzumab resulted in an increased expression of CCL5, demonstrating the crucial involvement of CCL5 in BC therapeutic resistance. However, the research conclusions were derived from animal experiments or in vitro cell models, lacking large sample prospective validation of tumor tissues from clinical trastuzumab-resistant patients, making it difficult to directly translate them into clinically applicable resistance prediction markers or intervention targets.

Thus, CCL5, functioning as a pivotal chemokine, demonstrates significant clinical application potential in early BC screening and disease progression prediction. Beyond its pivotal role in BC progression, CCL5 is also involved in promoting recurrence and treatment resistance through activation of the ERK signaling pathway. This indicates that CCL5 could serve as a target for inhibiting BC recurrence and reducing drug resistance, providing a crucial theoretical basis for establishing a CCL5-based molecular diagnostic system and targeted therapeutic strategies for BC.

## Therapeutic targeting of the CCL5/CCR5 axis in breast cancer

As previously discussed, the relationship between CCL5 and the onset and progression of BC is complex. Therefore, inhibiting the CCL5/CCR5 signaling axis within tumor tissues may represent a potential approach to suppress BC progression and metastasis [74].

### Combination immunotherapy

Studies have shown that oncolytic adenoviruses carrying the CCL5 gene could promote T cell infiltration into BC tissue. These viruses not only had oncolytic effects but also enhance the chemotaxis of T cells and other immune cells, resulting in a synergistic anticancer effect. This provided a new approach for the treatment of BC [75]. Lou et al. [76] further found that targeting and inhibiting CCL5 in combination with cryo-thermal therapy could lower the levels of MDSCs, while also promoting the phenotypic maturation of dendritic cells (DCs), inducing M1 macrophage polarization, regulating the functional differentiation of CD4^+^ T cells, and driving the production of cytotoxic CD8^+^ T cells. This alleviated tumor-driven immunosuppression and boosted antitumor immune activity, thereby enhancing treatment efficacy in invasive BC and prolonging survival in patients with aggressive disease.

### CCR5 antagonists

Currently, the CCL5/CCR5 axis has emerged as a promising therapeutic target for a range of cancers, including both solid tumors and hematological malignancies [74]. Studies have confirmed that CCR5 antagonists exhibit promising therapeutic effects in clinical cancer immunotherapy [77].

Common antagonists include Maraviroc and Verteporfin. Maraviroc blocks the interaction between CCL5 and CCR5, reducing the levels of key growth factors and cytokines in the tumor microenvironment that promote tumor growth and angiogenesis [78]. It also decreases the recruitment and polarization of TAMs and inhibits the EMT process, showing anti-metastatic effects in preclinical models [79]. In contrast, Verteporfin is more effective than Maraviroc in disrupting the CCL5/CCR5 axis. Verteporfin is a specific probe for the CCL5/CCR5 axis that selectively disrupts the CCL5/CCR5-YAP1 pathway, inhibits CCR5 expression, and further reduces YAP1 mRNA and protein expression via HIF-1α, significantly suppressing the growth and metastasis of TNBC [74]. Additionally, Lane et al. [80] found that lapatinib not only acted as an antagonist for CCR3 and CCR4 but also antagonized CCR5, suggesting the potential of lapatinib as a CCR5 antagonist.

In summary, combination immunotherapy and CCR5 antagonists effectively inhibit breast tumor development. Targeting the CCL5/CCR5 axis as a therapeutic strategy shows potential in enhancing the efficacy of BC treatment, offering new therapeutic avenues for BC. Although the intervention strategies targeting the CCL5/CCR5 axis have shown promising application prospects in many preclinical studies, retrospective cohort studies have preliminarily confirmed that CCL5/CCR5 may have potential as a diagnostic and prognostic biomarker for breast cancer. However, most existing relevant literature focuses on the exploration of basic mechanisms and lacks combined validation of basic experiments and large-scale clinical data. Together, these limitations form a barrier to translating CCL5/CCR5 findings from basic research to clinical use. In the future, it is urgent to conduct high-quality, multicenter, large-sample clinical studies and optimize preclinical animal models that better recapitulate human pathological features, so as to overcome translational bottlenecks and advance the clinical application of CCL5-related molecular targets in precision diagnosis and treatment of breast cancer.

## Research on CCL5 in antitumor immunity

Antitumor immunity is the process by which the immune system recognizes and eliminates tumor cells. While CCL5 mainly exerts a pro-tumorigenic functions in BC, in certain circumstances, increased CCL5 can promote the secretion of anticancer factors and enhance the infiltration of immune cells that have antitumor effects, thus generating an antitumor response.

Dangaj et al. [8] found that CCL5, as a key chemokine for CD8^+^ T cells, significantly enhanced T cell infiltration at the tumor site by cooperating with the expression of CXCL9, thus boosting the antitumor immune response and killing tumor cells. Ang [75] further demonstrated that CCL5 secreted by BC cells recruited T cells into the tumor, where tumor antigens activated them. The activated T cells produced IFN-γ, which in turn stimulated macrophages and DCs to secrete CXCL9, thereby enhancing circulating T cell infiltration and eliciting a robust antitumor immune response.

Sara Santana‑Hernández et al. [81] showed that neoadjuvant therapy with anti-HER2 antibodies activated tumor-infiltrating natural killer (TI-NK) cells to produce CCL5 and IFN-γ, and this IFN-γ triggered BC cells to secrete CXCL9 and CXCL10. CCL5, in cooperation with CXCL9 and CXCL10, directed TI-NK cells and T cells to infiltrate the solid tumor, remodeling a tumor-suppressive microenvironment and thereby triggering robust antitumor immune responses.

Schwarz et al. [82] found that combining Trabectedin and IL-12 treatment in TNBC stimulates NK cells in vivo to secrete chemokines CCL5 and XCL1, attracting type 1 conventional dendritic cells (cDC1) to the tumor microenvironment. The CXCL10 produced by cDC1 recruits CD8^+^ T cells, ultimately leading to elevated intratumoral CD8 + T cell infiltration and stronger antitumor activity.

Additionally, some researchers have shown that heat shock factor 1 (HSF1) restricted CD8^+^ T cell infiltration into breast tumors by lowering CCL5 expression, thereby inhibiting antitumor immune activity. The loss of HSF1 significantly upregulated CCL5, promoting CD8⁺ T cell infiltration and suppressing tumor growth. Therefore, targeting HSF1 might reprogram the tumor microenvironment to make it more immunogenic, thereby enhancing the response to standard BC therapies [83].

In summary, CCL5 possesses both pro-tumorigenic and anti-tumorigenic properties. The anti-tumorigenic effects included recruiting tumor-reactive tumor-infiltrating lymphocytes (TILs), which infiltrated tumors and destroyed tumor cells. On the other hand, the oncogenic properties of CCL5 lied in their ability to induce angiogenesis and lymphangiogenesis, recruited pro-tumor cells that supported cancer progression, and stimulated proliferation, migration, and invasion of cancer cells [84]. In growing tumors, the oncogenic properties of CCL5 were enhanced while its anti-tumor effects were suppressed. However, during the effective anti-cancer response of immunotherapy or the immune system, enhanced anti-cancer properties were displayed, which led to the infiltration of anti-cancer TILs into tumors and the destruction of tumor cells. In addition, an increase in the expression of a single chemokine did not always clearly indicate the prognosis of all cancer patients [85]. Studies have shown that CCL5 required CXCL9 as an “amplifier” for T cell recruitment, and co expression of CCL5 and CXCL9 could significantly promote T cell implantation and induce T cell-mediated immune responses [8].

**Fig. 3:**
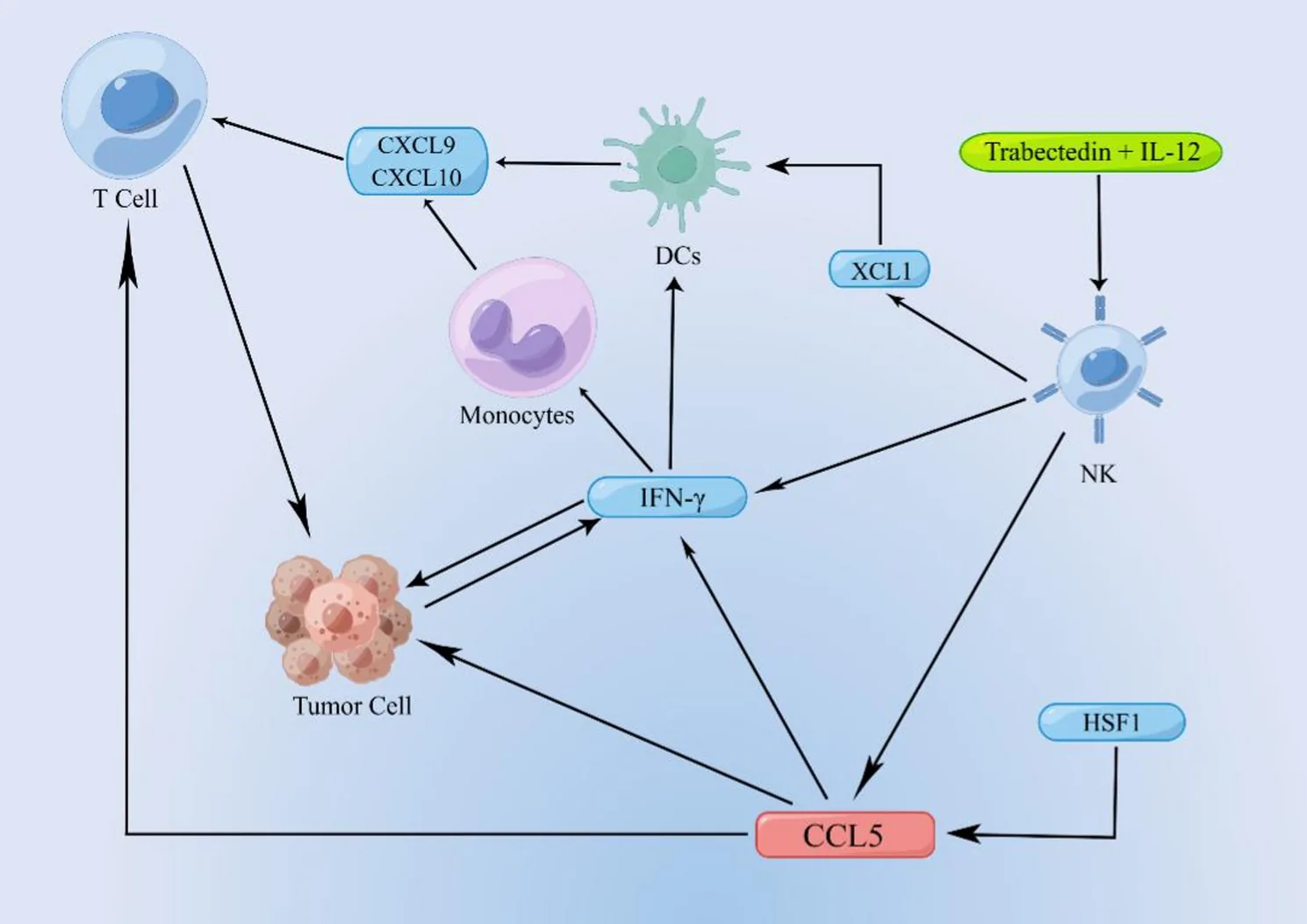
Context-dependent role of CCL5 in breast cancer antitumor immunity. In response to Trabectedin and IL-12, NK cells are activated and secrete XCL1 and IFN-γ, which stimulate DCs to produce CXCL9 and CXCL10. These chemokines recruit CD8⁺ T cells into the tumor microenvironment, where they exert cytotoxic effects and release IFN-γ, further amplifying immune activation. Notably, IFN-γ induces CCL5 expression in tumor cells, creating a positive feedback loop that enhances leukocyte recruitment. However, CCL5 expression can also be driven by the stress-responsive transcription factor HSF1, potentially linking therapy-induced stress to immune modulation. While CCL5 may support antitumor immunity by promoting immune cell infiltration, its net effect depends on the cellular context and cytokine milieu

Therefore, the final role of CCL5 in breast cancer also depends on the molecular subtype of the tumor (such as the difference between basal and luminal types), the immune status of the tumor microenvironment, the disease stage, the level and source of CCL5 in the tumor microenvironment, cell types with high CCR5 expression in the tumor microenvironment. Thus, it can be seen, CCL5 plays a paradoxical role in BC, exerting both tumor-promoting and tumor-suppressive effects. Its effects and mechanisms are complex, and further in-depth studies are needed to uncover the specific involvement of CCL5 in BC progression (Fig. 3).

## Conclusion and future perspectives

In summary, CCL5 appears to be an important regulatory factor in the BC microenvironment, activating multiple signaling pathways including PI3K/AKT, AMPK, and NF-κB. It is critically involved in BC proliferation, local invasion, distant metastasis, and post-treatment recurrence. The biological axis formed by CCL5 and its receptor CCR5 not only regulates the malignant phenotype of tumor cells but also shapes the immunosuppressive microenvironment by recruiting MDSCs, M2 macrophages, and Tregs, thus promoting BC progression. Given that CCL5 expression correlates positively with BC proliferation and migration, it has potential as a biomarker for early diagnosis and prognostic evaluation, providing more comprehensive evidence for selecting subsequent therapeutic strategies. Therefore, targeting the CCL5/CCR5 axis in tumor tissues to block the growth and metastasis of BC could be a promising anti-BC strategy. In future research, developing small molecule CCR5 antagonists and drugs that effectively inhibit CCL5 expression and secretion to interfere with the CCL5/CCR5 axis in BC patients could significantly improve survival outcomes and prognosis, offering broad clinical application prospects for BC treatment.

However, this review mainly centers on the CCL5/CCR5 axis, which is supported by sufficient cellular, animal and clinical research evidence in breast cancer. Only sporadic preliminary data about the CCL5/CCR3 axis related to tumor recurrence can be retrieved, and there are still prominent research gaps regarding its intact regulatory pathways and downstream molecular mechanisms. For other CCL5-binding receptors including CCR1, CCR4, ACKR1, ACKR2 and CCRL2, breast cancer-specific mechanistic research is extremely scarce at present. Sufficient published evidence is unavailable to support comprehensive and in-depth discussion of these receptors in the current work. Further targeted research focusing on CCL5 combined with CCR1, CCR3, CCR4, CCRL2 and ACKR family receptors is warranted as a promising direction for subsequent exploration.

Although CCL5 contributes markedly to BC advancement, some studies have indicated that CCL5 may also drive antitumor immunity under certain conditions. Thus, exploring additional downstream signaling pathways of CCL5/CCR5 and elucidating the specific molecular mechanisms of CCL5 in both promoting and inhibiting tumors will provide more theoretical foundations for targeting CCL5 in BC therapy.

Simultaneously, research has found significant species-specific preferences in the expression network of chemokines that drive tumor microenvironment TME assembly, the expression levels of CCR2 and CCR5 in mouse T cells are significantly reduced compared to humans, and there are fundamental differences in the intercellular communication and ecological network architecture that drive TME assembly between humans and mice [86]. Therefore, when studying the role of CCL5 in breast cancer, it is necessary to select models more carefully and deeply understand the tumor immune differences between species (Table 1).

**Table 1:** The roles and mechanisms of CCL5 in BC

	Upstream trigger	CCL5	Downstream trigger	Final biological effect	References
Proliferation	–	Overexpression of CCL5 in BC cells	Upregulation of Nanog expression in BC cells	Promoted BC cell proliferation	[17]
	–	LX-2 HSCs secreted CCL5	CCL5 bound to CCR5 receptors on BC cells; related signaling pathways were enriched in the PI3K/Akt pathway	Promoted the proliferation of BC cells	[18]
Invasion and metastasis	RANKL within the bone microenvironment induced expression of LTβ in tumor cells, LTβ bound to LTβR on osteoblasts, upregulated NF-κB p52, activated NF-κB2 signaling	Induced CCL2 and CCL5 production	CCL2/CCL5 recruited Raw264.7 macrophages, enhanced osteoclast differentiation	Promoted BC cell seeding in bone microenvironment and osteolytic bone metastasis	[25]
	S. aureus invaded BC cells and Activates NF-κB	NF-κB promoted CCL5 expression	CCL5 bound to the CCR5 receptor	The CCL5/CCR5 axis enhanced BC cell migration	[26]
	S. aureus invaded BC cells activates NF-κB p65 via RIG-I-like receptor signaling	Upregulated CCL5 and CCR5 expression	CCL5 bound to CCR5	Enhanced the migration capacity of BC cells	[27]
	Lactate secreted by BC cells acted on Notch1/Jagged2 receptors on the surface of TAMs, and upregulated the expression of NICD	Promoted the secretion of CCL5 and TGF-β; TGF-β further stimulated macrophages to secrete more CCL5	CCL5 bound to the CCR5 on BC cells, increasing the level of p-AMPK, and upregulated HK2 expression, and increases lactate production. Downregulation of E-cadherin, accompanied by upregulation of the mesenchymal markers vimentin and N-cadherin, indicated EMT in BC cells	Promoted aerobic glycolysis in BC cells, enhanced metastatic potential of BC cells and indicated lung metastatic	[30]
	Sulfur mustard exposure on MCF-7 BC cells	Enhanced CCL5 secretion and activated CCL5/CCR5 axis	upregulated Akt and p85α phosphorylation, activated the PI3K/Akt pathway Downregulated epithelial marker E-cadherin; upregulated mesenchymal markers FN mRNA, SNAIL2 and vimentin, induced EMT	induces EMT, promoted migration and invasion abilities of MCF-7 BC cells	[29]
	lncSNHG5 enhanced the stability of ZNF281	mediated CAFs to release CCL2/CCL5	CCL2/CCL5 reduced ZO-1 and Occludin levels in endothelial cells, then upregulated p-P38 expression in lung tissue	Increased microvascular density, enhanced lung capillary permeability. Promoted lung angiogenesis and formation of lung pre-metastatic niche	[33]
	–	CCL5 derived from D1 cells acted on BC cells	CCL5 bound to the CCR1 and CCR5 receptor on BC cells, induced secretion of MMP9, MMP13 and MMP14, MMPs degrade ECM proteins	Enhanced migration ability of BC cells, Promoted BC cell metastasis	[34, 35]
	Co-culture of MDA-MB-231 BC cells, adipocyte stem cells and skin-derived fibroblasts	Adipose-derived stem cells and MDA-MB-231 cells secreted high levels of CCL5	CCL5 induced the production of type VI collagen	Type VI collagen Promoted BC cell invasion and metastasis, accelerated cancer progression	[36]
	High-glucose environment, co-culture of TNBC cells and human adipocytes	Adjacent adipose tissue secreted CCL5	CCL5 triggered immune response	Facilitated lymphatic and distant metastasis, led to poor prognosis of TNBC	[37]
	High-glucose environment	promotes the secretion of CCL5 by adipocytes	CCL5 induced phosphorylation of AKT, mTOR and GSK3β, activated AKT/mTOR pathway, then Upregulated GLUT1 expression	Enhanced glucose uptake, elevated glycolytic flux and increased ATP production, enhanced the migration capacity of BC cells	[38]
Immune microenvironment	–	Stromal cells adjacent to BC cells expressed and secreted CCL5	CCL5 exerted chemotaxis to recruit CD163^+^ macrophages	Promoted BC progression and metastasis	[44]
	—	BC cells secreted CCL5	CCL5 bound to CCR5 on THP-1 monocytes, activated MEK/STAT3 pathway and induced phosphorylation of MEK1/2, ERK1/2 and STAT3, upregulated M2 macrophage markers (CD163, CD206, Arg1, IL-10). Monocytes polarize into M2 macrophages	–	[45]
	–	Primary breast tumor cells secreted CCL5	CCL5 bound to CCR5 on macrophages and increased p-Akt level to activate Akt pathway. Macrophages showed elongated morphology, increased CD206 and decreased HLA-DR, polarize into M2-type TAMs	–	[46]
	ZNF451 acted on BC cells	Slug (Snail2) mediated CCL5 transcription and expression	CCL5 recruits and activated TAMs, upregulated CD206, and macrophages undergo M2 polarization	Accelerated the progression of TNBC	[47]
	RKIP blocked HMGA2 signaling	Reduced the expression of CCL5 and other macrophage chemokines	CCL5 bound to CCR5	Inhibits TAM recruitment; CCL5 promoted TAM recruitment and infiltration in RKIP⁺ BC cells	[48]
	miR-9 overexpressed in 4T1 cells	miR-9 inhibited SOCS3 expression and upregulated CCL5	CCL5/CCR5 axis exerted chemotaxis on G-MDSCs, increasing proportion of G-MDSCs in tumor tissue, forming immunosuppressive microenvironment	Promoted BC proliferation	[54]
	–	Elevated CCL5 in tumor tissue of BC patients with lymphatic metastasis	CCL5 upregulated Treg markers CD25 and Foxp3 and recruits CD4⁺CD25⁺Foxp3⁺ Tregs, increasing Treg infiltration	Forms immunosuppressive microenvironment	[59]
	Tumor-derived OPN interacted with αvβ3 integrin on MSCs and activated transcription factor AP-1	promoted CCL5 secretion in MSCs	CCL5 induced MSCs to secreted α-SMA, tenascin-C, CXCL12 and FSP-1	MSCs transformed into CAFs	[62]
Recurrence	–	Stromal cells secreted CCL5	CCL5 bound to CCR3 on BC cells and activated downstream ERK pathway	Promoted tumor progression and increased BC recurrence risk	[44]
	Radiation therapy for TNBC	Increased CCL5 concentration	Raised blood monocyte levels, recruited and infiltrated macrophages. Macrophages polarized into immunosuppressive M2 subtype	Promoted tumor recurrence	[72]
	Continuous exposure of the drug-resistant BT-474 cells to trastuzumab	Increased expression of CCL5	Increased ERK phosphorylation	Mediated acquired resistance to trastuzumab; contributed to BC treatment resistance	[73]
Antitumor immunity	–	BC cells secreted CCL5	CCL5 recruits T cells into tumor, tumor antigens activate T cells. Activated T cells secreted IFN-γ, which induced macrophages and DCs to produce CXCL9	eliciting the antitumor immune response	[75]
	Neoadjuvant anti-HER2 antibody therapy	Activated TI-NK cells to secrete CCL5 and IFN-γ	IFN-γ induced BC cells to produce CXCL9 and CXCL10; CCL5 collaborates with CXCL9 and CXCL10. Promoted infiltration of TI-NK cells and T cells into tumor	Inhibited tumor progression and induced potent antitumor immune response	[81]
	Combined treatment with Trabectedin and IL-12 for TNBC	Stimulated NK cells to secrete CCL5 and XCL1	CCL5 and XCL1 recruited cDC1; cDC1 secreted CXCL10; CXCL10 recruited CD8⁺ T cells and increased their infiltration	Enhanced anti-tumor activity	[82]
	HSF1 was expressed in breast tumor tissue	HSF1 reduced CCL5 expression	Low CCL5 limited CD8⁺ T cell infiltration and inhibited anti-tumor immune activity; HSF1 loss elevated CCL5 and boosted CD8⁺ T cell infiltration	Enhanced tumor growth	[83]

In conclusion, research on the chemokine CCL5 holds significant potential, and further investigation into the interactions between CCL5 and BC will improve our insight into how BC progresses. This research also holds important clinical implications for BC diagnosis, treatment, and prognosis.

## Data Availability

No datasets were generated or analysed during the current study.

## Abbreviations

5-DFS: 5-year disease-free survival
ACKR1/DARC: Atypical Chemokine Receptor 1
ACKR2/D6: Atypical Chemokine Receptor 1
Akt: Protein kinase B
AMPK: AMP-activated protein kinase
BC: Breast Cancer
CA 15 − 3: Cancer antigen 15 − 3
CAFs: Cancer-associated fibroblasts
CCL2: C–C motif chemokine ligand 2
CCL5: C–C motif chemokine ligand 5
CCR1: C–C motif chemokine receptor 1
CCR3: C–C motif chemokine receptor 3
CCR4: C–C motif chemokine receptor 4
CCR5: C–C motif chemokine receptor 5
CCRL2/CRAM-B: Chemokine receptor-like protein 2
cDC1: Conventional dendritic cells
CDH1: E-cadherin
CM: Conditioned medium
CXCL12: Stromal cell-derived factor 1
DCs: Dendritic cells
ECM: Extracellular matrix
EGF: Epidermal growth factor
EMT: Epithelial–mesenchymal transition
ERK1/2: Extracellular signal-regulated kinase1/2
ER-α: Estrogen receptor
EVs: Extracellular vesicles
FGF: Fibroblast growth factor
FSP-1: Fibroblast-specific protein-1
GEO: Gene Expression Omnibus
G-MDSCs: Granulocyte-like myeloid-derived suppressor cells
GPCRs: G Protein-Coupled Receptors
HER2: Human epidermal growth factor receptor2
HK2: Hexokinase 2
HSCs: Hepatic stellate cells
HSF1: Heat shock factor 1
Jagged2: Jagged canonical notch ligand 2
LTβ: Lymphotoxin-β
LTβR: LTβ receptors
MDSCs: Myeloid-derived suppressor cells
MEK 1/2: Mitogen-activated protein kinase kinase 1/2
MMPs: Matrix metalloproteinases
MSCs: Mesenchymal stem cells
MVD: Microvascular density
NF-κB: Nuclear factor kappa-B
NICD: Notch intracellular domain
Notch1: Neurogenic locus notch homolog protein 1
OPN: Osteopontin
PI3K: Phosphoinositide 3-kinase
PMN: Pre-metastatic niche
PT: Primary breast tumor
p-AMPK: Phosphorylated AMPK
RANKL: Receptor activator of NF-κB ligand
RKIP: Raf kinase inhibitor protein
S. aureus: Staphylococcus aureus
siRNA: Small interfering RNA
STAT3: Signal transducer and activator of transcription3
TAMs: Tumor-associated macrophages
TCGA: The Cancer Genome Atlas
TGF-β: Transforming growth factor β
TILs: Tumor-infiltrating lymphocytes
TI-NK: Tumor-infiltrating natural killer
TME: Tumor microenvironment
TNBC: Triple-negative breast cancer
Tregs: T regulatory cells
VEGF: Vascular endothelial growth factor
WGCNA: Weighted Gene Co-Expression Network Analysis
ZNF: Zinc finger protein
α-SMA: α-smooth muscle actin
